# Real-Time Whole Genome Sequencing to Guide Patient-Tailored Therapy of Severe Acute Respiratory Syndrome Coronavirus 2 Infection

**DOI:** 10.1093/cid/ciac864

**Published:** 2022-11-03

**Authors:** Luke B Snell, Adela Alcolea-Medina, Themoula Charalampous, Christopher Alder, Tom G S Williams, Flavia Flaviani, Rahul Batra, Prijay Bakrania, Rajeni Thangarajah, Stuart J D Neil, Claire van Nispen tot Pannerden, Alina Botgros, Emma Aarons, Samuel T Douthwaite, Jonathan D Edgeworth, Gaia Nebbia

**Affiliations:** Department of Infection, Guy's and St. Thomas' NHS Foundation Trust, London, UK; Department of Infection, Guy's and St. Thomas' NHS Foundation Trust, London, UK; Department of Infection, Guy's and St. Thomas' NHS Foundation Trust, London, UK; Department of Infection, Guy's and St. Thomas' NHS Foundation Trust, London, UK; Department of Infection, Guy's and St. Thomas' NHS Foundation Trust, London, UK; National Institute for Health Research Biomedical Research Centre, Guy's and St. Thomas' NHS Foundation Trust, London, UK; Department of Infection, Guy's and St. Thomas' NHS Foundation Trust, London, UK; Department of Pharmacy, Guy's and St Thomas’ NHS Foundation Trust, London, UK; Department of Infection, Guy's and St. Thomas' NHS Foundation Trust, London, UK; Department of Pharmacy, Guy's and St Thomas’ NHS Foundation Trust, London, UK; Department of Infectious Diseases, King's College London, United Kingdom; Department of Infection, Guy's and St. Thomas' NHS Foundation Trust, London, UK; Department of Infection, Guy's and St. Thomas' NHS Foundation Trust, London, UK; Department of Infection, Guy's and St. Thomas' NHS Foundation Trust, London, UK; Department of Infection, Guy's and St. Thomas' NHS Foundation Trust, London, UK; Department of Infection, Guy's and St. Thomas' NHS Foundation Trust, London, UK; Department of Infection, Guy's and St. Thomas' NHS Foundation Trust, London, UK; Department of Infectious Diseases, King's College London, United Kingdom

**Keywords:** whole genome sequencing, COVID-19, SARS-CoV-2

## Abstract

The management of coronavirus disease 2019 has become more complex due to the expansion of available therapies. The presence of severe acute respiratory syndrome coronavirus 2 variants and mutations further complicates treatment due to their differing susceptibilities to therapies. Here we outline the use of real-time whole genome sequencing to detect persistent infection, evaluate for mutations confering resistance to treatments, and guide treatment decisions.

Coronavirus disease 2019 (COVID-19) treatments fall broadly into 2 categories: preemptive therapy in those at high risk of deterioration and treatments for hospitalized patients with acute illness [1]. The emergence of new variants and mutations can alter susceptibilities to these treatments, seen frequently with neutralizing monoclonal antibody (mAb) therapies [1]. Previously we have used severe acute respiratory syndrome coronavirus 2 (SARS-CoV-2) whole genome sequencing (WGS) to guide nosocomial outbreak investigation [2] and public health interventions [3]. We are now applying real-time WGS to characterize SARS-CoV-2 infection to detect persistent infection, evaluate for mutations confering resistance to treatments, and guide patient-tailored treatment decisions. The workflow takes 24 hours from sample receipt to results, allowing expedited treatment decisions. In this brief communication we present 6 cases where real-time WGS showed utility in guiding treatment.

## METHODS

WGS was performed on extracted nucleic acid from respiratory samples, using ARTIC v3.0 lab protocol with updated primer sets. After amplification, products underwent library preparation using SQK-LSK109, or SQK-RBK004 and run singleplex, on R.9.4.1 flow cells (Oxford Nanopore Technologies). Sequencing data were analyzed to call consensus sequences using the ARTIC bioinformatic protocol. Lineages were called using pangolin v2.0. To characterize mutations, genome annotation was performed using Systematic ProtEin AnnotatoR (SPEAR) v1.0. Analysis of variant viral populations was done using VarScan v2.0, as previously described [4]. Treatment was decided by a multidisciplinary team of virologists, infectious disease specialists, and pharmacists. Decisions outside national licensing and commissioning policies were authorized by the local Drugs and Therapeutics Committee. Note that in the United Kingdom (UK), COVID-19 therapies are centrally commissioned by government agencies. Case details and sequence results are summarized in Supplementary Table 1.

### WGS to Distinguish Chronic Infection From Reinfection

Preemptive therapy is advised for immunocompromised patients with acute infection at high risk of developing severe disease [1]. Chronic infection with SARS-CoV-2 can also occur in immunocompromised patients [5], and the limited evidence around its management suggests that patients may benefit from combination therapy and/or longer courses [6]. WGS can distinguish chronic infection from reinfection, by determining both the lineage assignment and nonlineage-defining single-nucleotide polymorphisms (SNP) [7]. Highly related genomes from longitudinal samples from 1 individual likely represent chronic infection rather than reinfection [7].

A 50-year-old woman (case 1) with a type BA thymoma had symptomatic COVID-19 confirmed by polymerase chain reaction (PCR) and lateral flow device (LFD) in February 2022, requiring admission and treatment with remdesivir and dexamethasone. She was discharged with a need for ambulatory oxygen, diagnosed as post–COVID-19 lung fibrosis with compatible imaging and lung function tests. She tested positive again in early April 2022; this was thought to represent shedding of nonviable RNA as it was within 60 days of symptoms. No WGS was available from samples taken between February and April 2022, as they were tested in another hospital. In June 2022, during workup for surgical resection of tumor at our hospital, she was LFD positive again, and reinfection was considered. However, WGS of this June 2022 sample identified the Omicron BA.1.1 sublineage, which had been eliminated from the UK for several months [8], strongly suggesting chronic infection continuing since initial illness in February 2022. As a result the patient was treated with dual agents: sotrovimab and Paxlovid (nirmatrelvir/ritonavir). The patient cleared infection with PCR-negative throat swabs for SARS-CoV-2 and underwent surgical resection of her tumor, and need for ambulatory oxygen resolved.

**Figure 1.**
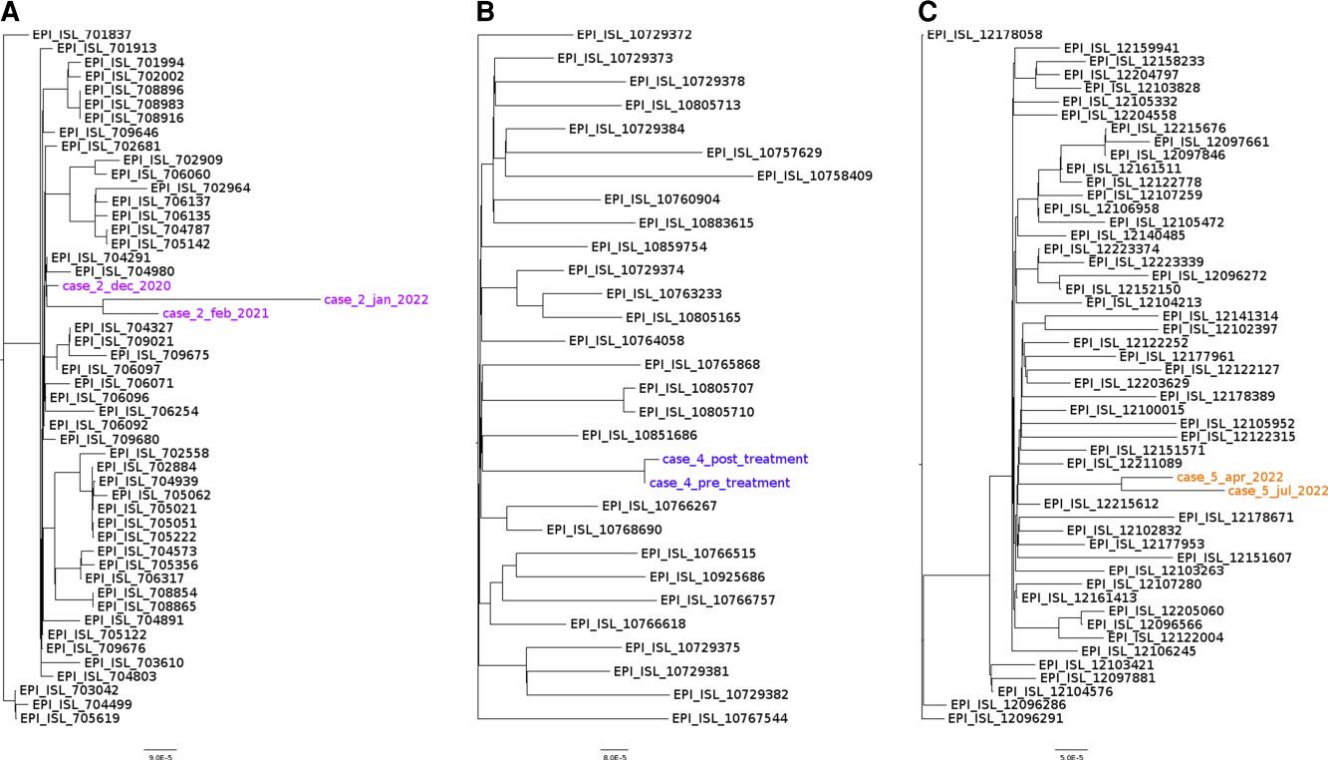
Phylogenetic representation of case 2 (*A*), case 4 (*B*), and case 5 (*C*). The outgroup for each case is represented by sequences from the same lineage from England, UK, submitted to GISAID in the same week as the case was diagnosed. A maximum of 50 sequences are displayed; where GISAID contains >50 sequences, subsampling was performed with seqtk v1.3. Maximum likelihood phylogenetic trees were computed using IQTree v.1.6, nodes calculated using ultrafast bootstrap and Shimodaira–Hasegawa approximate likelihood ratio test with 1000 replicates. Trees were visualized in FigTree v1.4.4. Branch tips are labeled with the GISAID accession number or case details.

One 59-year-old male outpatient (case 2) with kidney transplant and previous anti-CD20 treatment tested PCR positive for SARS-CoV-2 in December 2020, with WGS confirming lineage B.1.177.18. In February 2021 he tested positive again with WGS showing the same lineage B.1.177.18. The patient next returned in January 2022 and WGS again demonstrated SARS-CoV-2 B.1.177.18 lineage, differing by 18 SNPs from his original sample 13 months prior. Phylogeny of longitudinal sequence data are presented in Figure 1. This is consistent with chronic infection given the rate of mutation of SARS-CoV-2 is around 1 nucleotide every 2 weeks [9]. With ongoing paucisymptomatic, persistent infection the patient did not meet criteria for preemptive treatment, nor the treatment of hospitalized, acutely unwell patients. However, treatment was advised with REGN-COV2 (casirivimab/imdevimab) due to the presence of chronic infection with susceptible lineage. The patient cleared infection with PCR-negative throat swabs for SARS-CoV-2.

Similarly, WGS has also been used to confirm reinfection instead of chronic infection. A 44-year-old man (case 3) with a combined immunodeficiency was SARS-CoV-2 PCR positive in March 2022. He was treated with sotrovimab as per national guidelines for preemptive therapy [10]; he remained PCR positive on repeated testing including in May 2022 but became LFD negative. In June 2022 he had recrudescence of symptoms with fever and cough and LFD again became positive. His immunology team believed this represented recrudescence of persistent infection and proposed treatment with dual agents: sotrovimab and Paxlovid. WGS instead confirmed that episodes were caused by 2 separate infections with different sublineages of Omicron: BA.2, then reinfection with BA.5.2. As a result he received sotrovimab as preemptive treatment in line with commissioned guidelines, and dual therapy was not offered. The patient recovered.

### Evaluating for Mutations Conferring Reduced Susceptibility to Treatment

During chronic infection patients can receive several courses of treatment. COVID-19 therapies can lead to the emergence of mutations conferring resistance [1]. Establishing whether resistance mutations emerge after failed treatment can guide further rounds of therapy.

A 45-year-old woman (case 4) with advanced human immunodeficiency virus had chronic, asymptomatic SARS-CoV-2 Delta AY.4 infection identified in February 2022. She was treated with REGN-COV2 (casirivimab/imdevimab). WGS of a posttreatment sample taken 19 days after treatment showed emergence of a G22989A mutation below consensus level but in the majority of reads. This confers a spike G476D substitution, known to reduce sensitivity to casirivimab by 1021-fold [11]. Similarly, T22896C was seen in a majority of reads spanning this position, conferring spike V445A and a >500-fold decrease in sensitivity to imdevimab [11]. WGS of the pretreatment sample showed wild-type sequence at these positions. There are limited data on the neutralization of Delta by sotrovimab [12] and some data that this treatment can lead to rapid resistance [13]. This information, along with the development of resistance to REGN-COV2, supported the decision to forgo further treatment with neutralizing mAbs. Instead, a course of Paxlovid was offered. In August 2022, her throat swabs became SARS-CoV-2 PCR negative after immune reconstitution.

Similarly, a 60-year-old man (case 5) was immunocompromised from previous treatment with obinutuzumab for chronic lymphoid leukemia. He developed COVID-19 in April 2022 and was later admitted with COVID-19 pneumonitis and respiratory failure. After receiving Paxlovid and double-dose (1000 mg) sotrovimab in mid-July, he had higher cycle threshold values in his throat swabs, showed clinical improvement, and was discharged. However, approximately 6 weeks after treatment he was readmitted with hypoxia and progression of pneumonitis. Other infectious etiologies were excluded and respiratory sampling confirmed a high SARS-CoV-2 viral load. Persistent BA.2.3 infection was confirmed by comparison of longitudinal sequence, with only 7 SNPs difference over 10 weeks of infection. Sequencing of his July 2022 sample also revealed posttreatment emergence of a spike K356R substitution. This substitution can arise after treatment with sotrovimab [14] but does not affect the in vitro activity of sotrovimab [15]. In addition, resistance to Paxlovid was investigated by looking at the sequence of the nsp5 gene, which encodes the main protease of SARS-CoV-2, which Paxlovid targets [1]. Only the Omicron lineage-defining P132H was detected, and in vitro evidence suggests this does not affect inhibition by Paxlovid [16]. No mutations associated with resistance to remdesivir were identified in the RNA-dependent RNA polymerase encoded by nsp12, which is the target of remdesivir [1, 17]. This information supported the decision to retreat with Paxlovid and remdesivir. Outcome is awaited.

### Determination of Lineage to Direct Neutralizing mAb Therapy

Sublineages of Omicron have varying in vitro susceptibility to sotrovimab [18], which is the only neutralizing mAb licensed in the UK with activity against Omicron. Sotrovimab has most activity against BA.1, reduced against BA.4/5, and least activity versus BA.2 [18]. Subsequently, clinical trials are evaluating double-dose (1000 mg) sotrovimab for Omicron sublineages such as BA.2 with decreased susceptibility [19]. We have given double-dose sotrovimab to cases where WGS confirms PCR genotyping results that suggest Omicron sublineages are present with decreased susceptibility. Notably, our PCR-based genotyping assay [20] cannot distinguish between emerging sublineages of Omicron, meaning WGS may have increasing value for identifying resistant variants.

A 47-year-old immunocompromised woman (case 6) with primary sclerosing cholangitis awaiting liver transplantation developed hospital-onset COVID-19. BA.5 was dominant in London at this time, but WGS identified the BA.2.12.1 variant. Given the sublineage detected, double-dose sotrovimab was recommended as preemptive therapy—the patient being ineligible for other commissioned therapies (Paxlovid and remdesivir) due to interactions and contraindications, respectively. The patient recovered with SARS-CoV-2 PCR–negative throat swabs.

## CONCLUSIONS

Whole genome sequencing has utility in guiding treatment decisions, especially in complex cases with chronic infection. These cases illustrate the benefit of characterizing emergent resistance after treatment, and in detecting SARS-CoV-2 variants below consensus frequency in the viral population. Of note, this method cannot exclude the presence of low-frequency minority variants that could confer resistant phenotypes. To this end, workflows able to detect low-frequency, resistant minority variants may be required. Elucidation of mutations associated with resistance will be important for maximum utility of sequencing-directed therapy. In our experience, rapid, near-patient capability for WGS, analysis, and interpretation were important to guide patient-tailored therapy for COVID-19. Evidence on optimal treatment of chronic infection is also needed.

## Supplementary Data

Supplementary materials are available at *Clinical Infectious Diseases* online. Consisting of data provided by the authors to benefit the reader, the posted materials are not copyedited and are the sole responsibility of the authors, so questions or comments should be addressed to the corresponding author.

## Notes

***Data availability.*** Consensus sequences and read data are available on the Sequence Read Archive (BioProject: PRJNA881323). Individual National Center for Biotechnology Information genome accessions: OP680467–OP680475.

***Ethics statement.*** Collection of surplus samples and linked clinical data was approved by South Central—Hampshire B REC (20/SC/0310). Treatment decisions outside of licence and/or commissioning policy were agreed by both the multidisciplinary team and local Drug and Therapeutics Committee.

***Financial support.*** L. B. S. and G. N. have received grants from the Medical Research Council (MRC) (MR/W025140/1; MR/T005416/1). A. A. -M., T. C., L. B. S., G. N. and R. B. report grants from the MRC Confidence in Concept (grant reference: MC PC 19041).

## Supplementary Material

ciac864_Supplementary_Data
